# Supplementary material for: Comparing synthetic refocusing to deconvolution for the extraction of neuronal calcium transients from light fields
Source: Neurophotonics. 2022 Mar 11;9(4):041404. doi: 10.1117/1.NPh.9.4.041404 (PMC8922050; doi:10.1117/1.NPh.9.4.041404)
Supplement: Supplementary file 1 [file NPh_009_041404_SD001.pdf]

Comparing synthetic refocusing to deconvolution for the extraction of neuronal calcium transients from light-fields

Carmel L. Howe[a,b], Peter Quicke[a,b], Pingfan Song[c], Herman Verinaz Jadan[c], Pier Luigi Dragotti[c], Amanda J. Foust[a,b,*]
[a]Department of Bioengineering, Imperial College London, London, UK
[b]Centre for Neurotechnology, Imperial College London, London, UK
[c]Department of Electrical and Electronic Engineering, Imperial College London, London, UK
*Amanda J.Foust, E-mail: a.foust@imperial.ac.uk

S1 Comparison of Richardson-Lucy 3D Deconvolution with and without Total Variation (TV) regularization

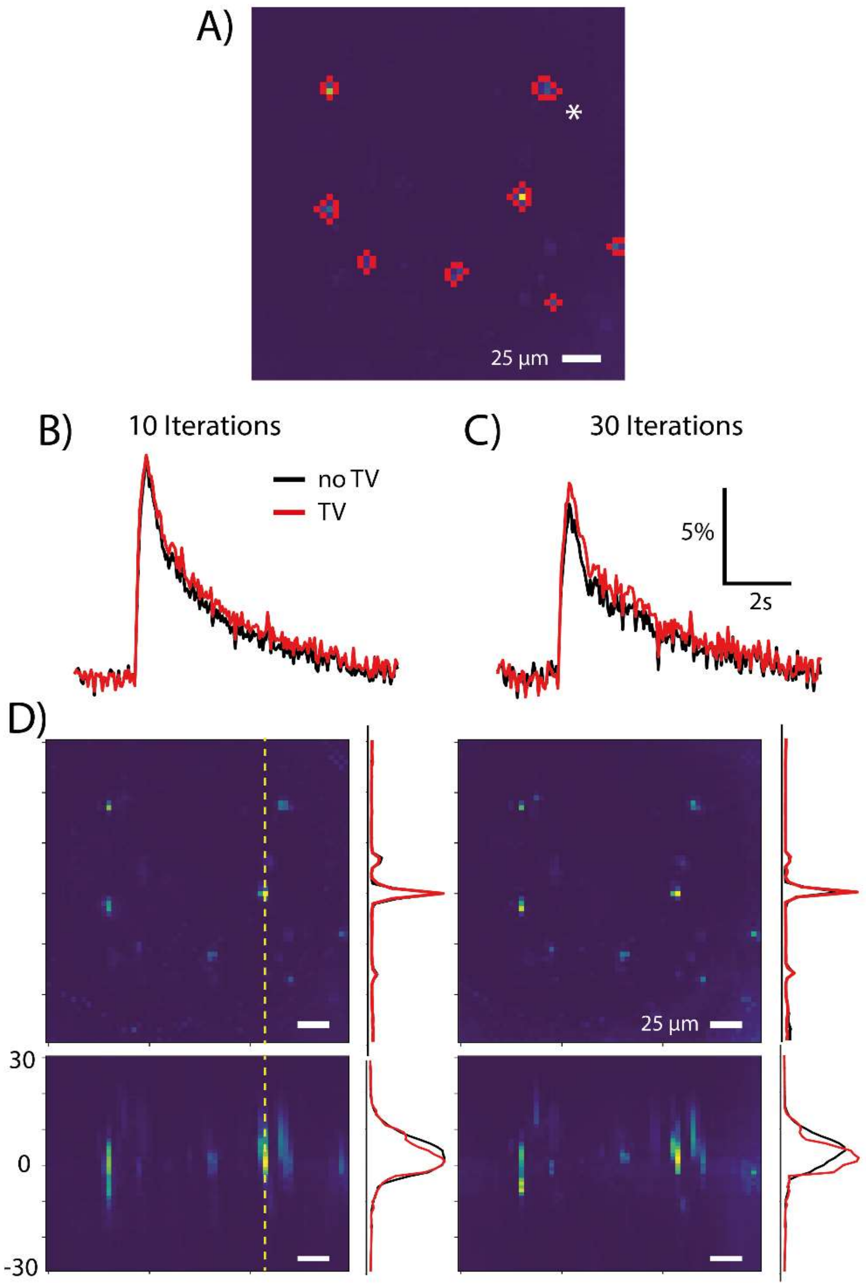

Fig. S1 (a) Raw reconstructed light-field volumes have low contrast therefore, an activation map was generated from the variance over time to resolve active neurons and shown as a maximum intensity projection through z. Active neurons are indicated with a red outline. **(b,c)** Calcium time series from one neuron (indicated with asterisk);

reconstructed with RL deconvolution with and without the temporal variation term after 10 **(b)** and 30 **(c)** iterations. **(d)** Planes from bulk-labeled slices were reconstructed from light-field volumes reconstructed with RL deconvolution with and without total variation regularization between -30 and +30 um in steps of 1 μm. A maximum intensity projection was generated, and a line profile taken from the indicated slices.

All scale bars are 25 μm.

S2 Videos

S2A – Time series of synthetically refocused (left) and 3-iteration RL 3D deconvolved light-fields (right) from a single CaSIR-1-labeled cell at the native plane of focus. Video is at 7 fps. Scale bar is 25 μm.

S2B – Z-stack of widefield (left, with pipette removed), and 3-iteration RL 3D deconvolution reconstructed light fields from a static, single-labeled cell (center), and the functional activation map of dF/F variance over the time-series (right). Volumes are shown between -30 and +30 μm. Video is at 7 fps. Scale bars are 25 μm.
